# Laryngeal Sensitivity in Patients with Amyotrophic Lateral Sclerosis

**DOI:** 10.3389/fneur.2016.00212

**Published:** 2016-11-28

**Authors:** Giovanni Ruoppolo, Emanuela Onesti, Maria Cristina Gori, Ilenia Schettino, Vittorio Frasca, Antonella Biasiotta, Carla Giordano, Marco Ceccanti, Chiara Cambieri, Antonio Greco, Costantino Eugenio Buonopane, Giorgio Cruccu, Marco De Vincentiis, Maurizio Inghilleri

**Affiliations:** ^1^Department of Sensorial Organs, Sapienza University, Rome, Italy; ^2^Department of Neurology and Psychiatry, Sapienza University, Rome, Italy; ^3^Department of Radiological Sciences, Oncology and Anatomical Pathology, Sapienza University, Rome, Italy; ^4^Intensive Care Service, Emergency Department, Sapienza University, Rome, Italy

**Keywords:** larynx, amyotrophic lateral sclerosis, deglutition disorders, sensory nerve endings, PGP 9.5

## Abstract

Recent studies have shown the involvement of the sensory nervous system in patients with amyotrophic lateral sclerosis (ALS). The aim of our study was to investigate the correlation between the laryngeal sensitivity deficit and the type of ALS onset (bulbar or spinal) in a large series of 114 consecutive ALS patients. Participants were subdivided into two groups, bulbar and spinal ALS, according to the clinical onset of disease and submitted to a clinical and instrumental evaluation of swallowing, including a fiber-optic endoscopic evaluation of swallowing with sensory testing. Dysphagia severity was scored using the Penetration–Aspiration Scale (PAS) and the Pooling score (*P*-score). In addition, three patients with laryngeal sensitivity deficit were submitted to a laryngeal biopsy to assess the status of the sensory innervation. All patients showed a normal glottal closure during phonation and volitional cough. Fifty-six subjects (49%), 14 spinal- and 42 bulbar-onset ALS, showed dysphagia at the first clinical observation (PAS score >1; *P*-score >5). Dysphagia resulted more frequently in bulbar-onset ALS (*P* < 0.01). Thirty-eight (33%) patients had a sensory deficit of the larynx. The sensory deficit of the larynx was significantly more frequent in bulbar-onset ALS (*P* < 0.01). The sensory deficit of the larynx among dysphagic patients was also significantly more frequent in bulbar-onset ALS (*P* = 0.02). Several abnormalities were found in all three subjects who underwent a laryngeal biopsy: in one patient, no intraepidermal fiber was found; in the other two, the fibers showed morphological changes. Our observations are important to consider for assessment and management of dysphagia in patients with ALS.

## Introduction

Amyotrophic lateral sclerosis (ALS) is a neurodegenerative disease causing progressive physical disabilities due to deficits of the motor nervous system. The upper and lower motor neurons are primarily affected, but involvement of the sensory nervous system has also been reported (1). In a multicentre, large electrophysiological study on 88 patients with ALS, abnormalities in the sensory nerve conduction parameters were found in 22.7% of the subjects (2). Sensory involvement in 32% of 103 ALS patients was also found in another electrophysiological study and confirmed by evidence of sensory nerve pathology in 91% of the 22 patients additionally subjected to sural-nerve biopsy (3). In recent years, immunohistochemistry, by means of a selective pan neuronal marker used to highlight peripheral nerve tissue, the pan-axonal anti-protein-gene-product 9.5 (PGP 9.5), has emerged as a valuable tool to analyze sensory and autonomic innervation (4–6). Using this immunohistochemical technique, a significant reduction was found in epidermal nerve fiber density in the distal calf of 28 patients with ALS, supporting the concept of distal axonopathy in ALS (1). In a recent study, skin biopsies taken both from the thigh and from the distal leg of ALS patients disclosed normal intraepidermal nerve fiber density (INFD) in patients with bulbar onset, and a reduced INFD in patients with spinal onset (7). Sensorial deficits were recently found also at the laryngeal level. In a study on swallowing function in ALS, performed by means of fiber-optic endoscopic evaluation of swallowing (FEES) with sensory testing, abnormal sensation was found in 54.5% of the 22 tested patients (8). In a study on prevalence of dysphagia in ALS, an impaired laryngeal adduction reflex (LAR) resulted in 20.4% of the 49 ALS patients studied (9). Although the major factor causing swallowing troubles in ALS patients is the tongue muscle deficit, which correlates with pooling and penetration (9), the abnormal sensation of the larynx can be regarded as a crucial factor in worsening dysphagia, as it reduces the efficacy of the LAR, consequently decreasing lung protection. The LAR, a brief closure of the true vocal folds, is a sensorimotor response that plays a key role in defending the lower airways during swallowing and protecting the larynx from food or fluid aspiration during premature spillage of material from the oral cavity or post-swallow inspiration. By contrast with the reflex control of breathing, volitional cough is probably mediated through corticobulbar pathways. The upper and lower motor neuron contribution to various respiratory muscles can be assessed neurophysiologically using magnetic stimulation of the motor cortex and spinal roots (10).

The aim of our study was to investigate the correlations between laryngeal sensitivity deficit and the type of ALS onset (bulbar or spinal) in a large series of ALS patients. In addition, three patients with laryngeal sensitivity deficit were submitted to laryngeal biopsy to assess directly the condition of the sensory innervation. The laryngeal biopsies were obtained during the tracheostomy performed for severe respiratory failures. The finding of a correlation between the sensitivity of the larynx and the type of ALS could shed new light on the pathophysiology of ALS and could have potential implications on clinical diagnosis and on the treatment of dysphagia.

## Materials and Methods

A total of 114 ALS patients, diagnosed according to the El Escorial criteria at the ALS Center, Department of Neurology and Psychiatry, Umberto I Hospital, University of Rome “Sapienza,” were enrolled retrospectively. The study sample comprised 42 women and 72 men (mean age 64 ± 10 years, range 42–82 years). Bulbar onset was reported in 56 patients and spinal onset in 58 patients. The mean ages of the two groups were similar (66.2 ± 8.6 in bulbar, 61.5 ± 9.9 in spinal ALS).

All patients were submitted to a clinical and instrumental evaluation of swallowing at the first admission into the ALS Center, by means of FEES, using a 3.7-mm diameter flexible fiber-optic rhinolaryngoscope (Storz 11101). Dysphagia, if present, was classified as “oral,” “pharyngeal,” or both (“oropharyngeal”). The study of laryngeal sensitivity was based on lightly touching the pharyngeal walls, the laryngeal surface of the epiglottis, the aryepiglottic folds, or the arytenoids, using the tip of the probe according to the Langmore protocol (11). Laryngeal sensitivity was judged as pathologic when touching the epiglottis failed to trigger the LAR (12). Finally, to verify the functionality of the laryngeal adductor muscles, all the subjects were requested to phonate the vowel “i” and to voluntarily cough throughout a fiber-optic examination. Dysphagia severity was classified both according to aspiration events with the Penetration–Aspiration Scale (PAS) (13) and considering the amount and the ability to control residue/bolus pooling using the Pooling score (*P*-score) (14). Clinical assessment was also conducted with the revised ALS Functional Rating Scale (ALSFRS-R), a validated measure of functional impairment in ALS (15) containing 12 questionnaire-based items rated from 0 (complete dependence for that function) to 4 (normal function), divided into three subscores (bulbar 12, motor 24, and respiratory 12), with normal function defined by a score of 48. Muscle strength of lower limbs was assessed with the Medical Research Council (MRC) scale for grading muscle strength, ranging from 0 (absence of movement) to 5 (contraction against full resistance), that quantifies muscle weakness in isolated muscles or muscle groups (16). In the MRC test, eight muscle groups in upper limbs and seven muscle groups in lower limbs are tested. The maximum scores are 40 for each upper limb and 35 for each lower limb.

Furthermore, in three subjects in whom FEES failed to elicit the LAR, an epiglottis biopsy specimen was also excised, immediately after tracheotomy, under general anesthesia, by means of microlaryngoscopy. Samples were fixed overnight in Zamboni’s solution and cryoprotected in 20% sucrose phosphate-buffered saline solution, cut into 50-μm-thick sections on a freezing slide microtome (Leica 2000R; Leica Microsystems, Wetzlar, Germany). Three sections randomly chosen from each biopsy were immunoassayed with polyclonal anti-PGP 9.5 antibodies (1:1000; Biogenesis), using a free-floating protocol for bright-field immunohistochemistry. The linear density of the intraepitelial nerve fibers was calculated according to the guidelines of the European Federation of Neurological Societies/Peripheral Nerve Society (17). Skin biopsy results were compared with the values found in our previous study on nerve endings in human laryngeal mucosa (12). The study was performed according to the ethical standards laid down in the Declaration of Helsinki, written informed consent was obtained from all research participants.

### Statistical Analysis

Summary statistics are presented as frequencies and percentages, mean ± SD. The Yates-corrected chi-square test was used to compare qualitative values. A *t*-test was used to compare means in normal groups. Statistical significance was accepted at *P* < 0.05. The delay between the onset of symptoms and the beginning of dysphagia at the date of last follow-up was measured with Cox’s *F*-test.

## Results

All the clinical and instrumental examinations were performed 14 ± 7.5 months after clinical onset (range 1–36 months). The delays between the onset of symptoms and the inclusion in the study were similar in both the spinal- and bulbar-onset ALS patient groups (12 ± 6.6 months in bulbar ALS, 12.2 ± 8 months in spinal ALS). Overall, the PAS score, the *P*-score and the ALSFRS-R score were respectively 4.7 ± 2.6, 8 ± 2.3, and 39 ± 6.2. All the patients showed a normal glottal closure during phonation and volitional cough.

**Figure 1:**
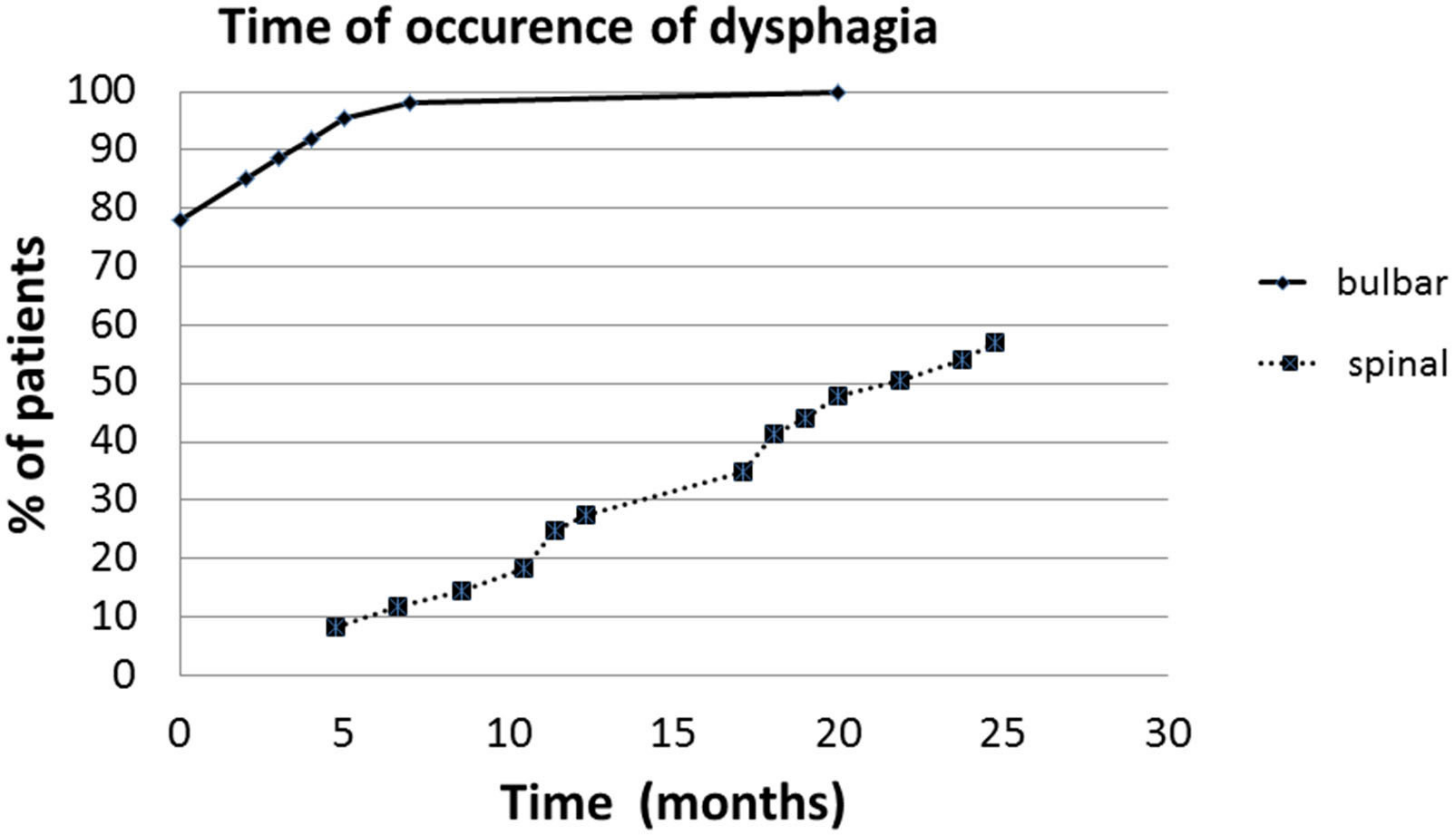
**The delay between the onset of symptoms and beginning of dysphagia in spinal and bulbar ALS (months)**.

Sixty-six subjects (58%; 18 spinal and 48 bulbar) showed dysphagia at the first clinical observation (PAS score >1; *P*-score >5). Dysphagia was more frequent in bulbar-onset ALS (*P* < 0.01) (Table 1). PAS score and *P*-score were significantly higher in bulbar-onset ALS (PAS score = 4.8 ± 2.6; *P*-score = 8 ± 2.3) than in spinal onset (PAS score = 1.8 ± 1.5; *P*-score = 5 ± 2) (*P* < 0.01). The impairment of both swallowing phases detected by FEES was more frequent in bulbar ALS (*P* < 0.01): 40 of the 44 subjects with oropharyngeal dysphagia had bulbar-onset ALS (Table 2). No patient showed, exclusively, impairment of pharyngeal phase. At the retrospective analysis, the onset and the evolution of dysphagia were different in the bulbar and spinal groups (*F* = 6.6; *P* < 0.01) (Figure 1).

**Table 1:** **ALS patients with or without dysphagia at baseline according to the spinal or bulbar onset**.

	Bulbar	Spinal	Total
Dysphagia	48 (42%)	18 (16%)	66 (58%)
No dysphagia	8 (7%)	40 (35%)	48 (42%)
Total	56 (49%)	58 (51%)	114

*The results are expressed as absolute numbers and percentages. Dysphagia was evaluated by PAS score >1 and P-score >5*.

**Table 2:** **Impairment of the swallowing phases detected by FEES in bulbar and spinal ALS patients**.

	Oral	Pharyngeal	Oral + pharyngeal	Total
Bulbar	8 (12%)	0 (0%)	40 (61%)	48 (73%)
Spinal	14 (21%)	0 (0%)	4 (6%)	18 (27%)
Total	22 (33%)	0 (0%)	44 (67%)	66

Of the 114 patients enrolled, 38 subjects (33%) had a larynx sensory deficit: 34 had bulbar ALS, while 4 had spinal onset (Table 3). The sensory deficit of the larynx was significantly more frequent in bulbar ALS (*P* < 0.01). All patients with a larynx sensory deficit were also dysphagic: among the 56 patients with dysphagia, 38 subjects (68%) showed abnormal laryngeal sensitivity, while 18 (32%) had normal sensitivity of the larynx. The sensory deficit of the larynx among dysphagic patients was significantly more frequent in bulbar ALS (*P* = 0.02). Specifically, 34 of the 38 subjects with dysphagia and abnormal laryngeal sensitivity had bulbar ALS, while only 4 had spinal onset. The clinical and instrumental characteristics of the two groups, normal and abnormal laryngeal sensitivity, are reported in Table 4.

**Table 3:** **Laryngeal sensitivity in relation to ALS onset**.

	Bulbar	Spinal	Total
Laryngeal sensory deficit	34 (30%)	4 (4%)	38 (33%)
Normal laryngeal sensitivity	22 (19%)	54 (47%)	76 (67%)
Total	56 (49%)	58 (51%)	114

*Laryngeal sensitivity was considered abnormal when the laryngeal adductor reflex (LAR) was not triggered with the touch of the epiglottis. All patients with abnormal LAR were dysphagic*.

**Table 4:** **Clinical and instrumental variables in the two groups, with normal laryngeal sensitivity and laryngeal sensory deficit**.

	Laryngeal sensitivity deficit	Normal laryngeal sensitivity	*P*
Age	67 ± 10	63 ± 10	ns
Delay between the onset of ALS symptoms and clinical observation	11 ± 7	15 ± 7.5	ns
ALSFRS	37 ± 8	38 ± 7.8	ns
PAS	6 ± 2.4	2 ± 1.8	<0.01
*P*-score	8.6 ± 2.2	5 ± 2	<0.01
FVC	2.3 ± 1	3 ± 1	ns
FEV1	1.9 ± 0.9	3.1 ± 2.5	ns
FEV1/FVC	79 ± 24	85 ± 10	ns
MRC	137 ± 12	127 ± 28	ns

*The results are expressed as mean and SD*.

*ns, not significant; FVC, forced vital capacity; FEV1, forced expiratory volume at one second; FEV1/FVC, Tiffeneau index*.

The ALSFRS scores were similar between the two groups, with slightly lower values for the group with a sensory deficit of the larynx (37 ± 8 vs. 38 ± 7.8). The PAS scores were different between the two groups: the group with laryngeal sensory deficit had higher scores than the normal sensory group (6 ± 2.4 vs. 2 ± 1.8; *P* < 0.01). The *P*-scores were also significantly different between the two groups, with higher scores for the group with a sensory deficit of the larynx (8.6 ± 2.1 vs. 6 ± 1.1; *P* < 0.01). Patients without laryngeal sensory deficits were more likely to have dysphagia involving only the oral phases (18 of the 22 patients with impairment of the oral phases showed a normal laryngeal sensitivity), while patients with sensory deficits of the larynx were more likely to display oropharyngeal dysphagia (34 of the 44 with an impairment in both the phases of swallowing showed a laryngeal sensory deficit).

With regard to the subjective symptoms of dysphagia, 52 patients (46%) reported swallowing problems at the first admission into the ALS Center, confirmed by a PAS score >1 and a *P*-score ≥6. Sixty-two patients (54%) did not refer dysphagia. In 58 of them, normal swallowing was confirmed by a PAS score = 1 and a *P*-score ≤5, while in 4, the swallowing examination showed an impairment of the oral phases of swallowing (PAS score >1 and *P*-score ≤5), with a larynx sensory deficit in 2.

**Figure 2:**
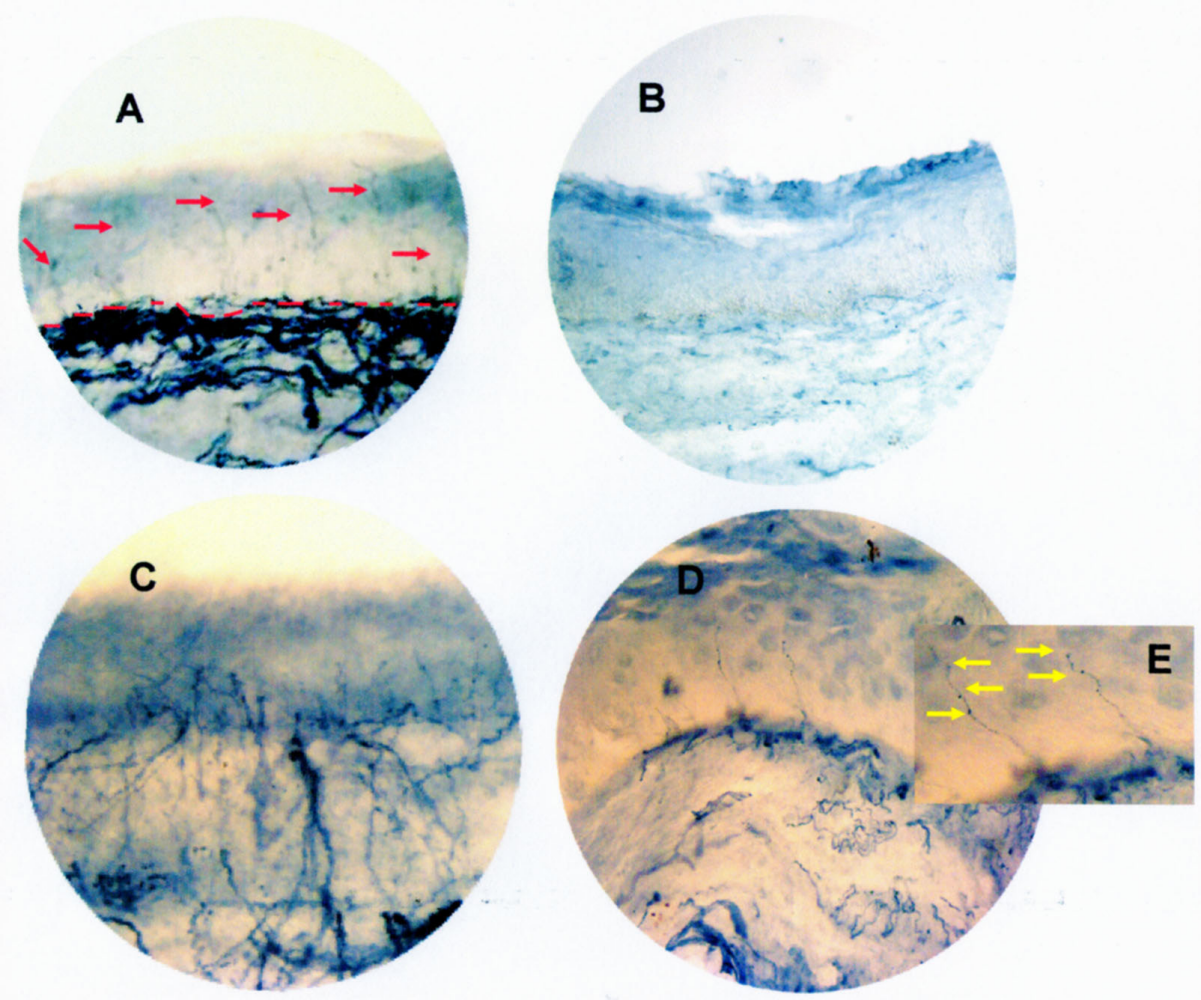
**Mucosa biopsy section from the epiglottis site of a control subject (red arrows show nerve fibers; red dotted lines indicate the junction between epithelium and lamina propria) (A), of the first ALS patient (showing the lack of intraepithelial fibers in the entire thickness of the epithelium) (B), of the second ALS patient (showing an increased and chaotic nerve branching not crossing the whole epithelium) (C), and of the third ALS patient (showing large axonal swellings in many branches of the intraepithelial fibers) (D)**. In the enlarged image **(E)**, yellow arrows point to the axonal swelling. All three ALS patients were dysphagic and had a laryngeal sensory deficit. Bright-field immunohistochemistry with polyclonal PGP 9.5 antibody. Original magnification 40×.

All three patients who underwent a laryngeal biopsy were dysphagic and had a laryngeal sensory deficit. Several abnormalities were found in all three subjects compared to the control subject (Figure 2): in the first patient, no intraepithelial fibers were found (Figure 2B vs. Figure 2A); in the other two patients, the number of fibers (respectively, 1.2 and 0.9/mm) was in the range of density obtained in our previous observations on normal subjects (0.83 ± 0.51/mm) (12), but the fibers showed clear morphological changes. In the second patient, the nerve branching was increased and chaotic; moreover, the fibers did not cross the whole epithelium (Figure 2C vs. Figure 2A). In the third patient, we observed large axonal swellings in many branches of the intraepithelial fibers (Figures 2D,E vs. Figure 2A).

## Discussion

In our study, a sensory deficit of the larynx was found in 33% of ALS patients, by means of the sensitivity test performed during FEES. The sensory deficits of the larynx were significantly more frequent in subjects with bulbar-onset ALS than in patients with spinal onset. All the patients of our sample showed a normal glottal closure during phonation and volitional cough, thus assuring us that the failure in LAR triggering was not related to a motor deficit. In three patients, the presence of a sensory deficit at the laryngeal level was also confirmed by laryngeal biopsies performed during tracheostomy. While in one patient, no intraepithelial fiber was found, the other two showed morphological changes, such as increased branching and axonal swellings. Axonal swellings have been described either quantitatively as enlargements above 1.5 μm (18) or semiquantitatively as enlargements more than twice the diameter of the parent fiber (19, 20). This finding is most commonly considered a predegenerative change. Increasing branching and axonal swellings have been frequently observed in patients with neuropathy and might represent an early sign of nerve fiber dysfunction (21, 22).

Dysphagia (PAS score >1; *P*-score >5) was found in 56 subjects (49%), and it was significantly more frequent in subjects with bulbar-onset ALS. The onset of symptoms was reported by the patients and/or caregivers and could be underestimated due to the patients’ ability to adapt to the difficulties in swallowing. As expected, a large number (68%) of dysphagic ALS subjects displayed also a deficit of laryngeal sensitivity, and all the subjects with a laryngeal sensitivity deficit were also dysphagic. The sensory deficit of the larynx among dysphagic patients was observed significantly more frequently in bulbar ALS. However, about one-third of the dysphagic subjects (32%) showed a normal LAR. These data are probably due to the peculiar progression of swallowing deficits in ALS patients, involving the oral phases earlier. In our sample, the impairment of the pharyngeal phase was always observed in association with a deficit of the oral phases, and 89% of dysphagic patients with laryngeal sensory deficits displayed dysphagia involving also the pharyngeal phase. In our opinion, in ALS patients, already suffering from oropharyngeal or pharyngeal dysphagia due to the reduced muscular efficiency of the tongue base and of the pharynx, the sensorial failure in triggering the LAR could be responsible for a further worsening of the pharyngeal phase of swallowing. At present, however, sensory deficits are not specifically described in swallowing scales and the classification of the dysphagia severity releases only on the level of bolus penetration, according to the widely accepted PAS (13).

Our data are consistent with several previous studies suggesting that ALS is a neurodegenerative pathology not confined to motor neurons but also affecting sensory neurons. In a previous study, we showed that, in patients with spinal-onset ALS, distal INFD at the skin biopsy of the leg was reduced. Conversely, in patients with bulbar-onset ALS, skin biopsy of the leg disclosed normal INFD (7). Sensory involvement in ALS patients has been demonstrated by means of subjective descriptions, electrophysiological recordings, and pathologic studies (1–7). In particular, Weis et al. (1), exploring the prevalence of small fiber neuropathy in ALS patients with the same immunohistochemical technique we used, found a significant reduction of INFD in the distal calf of ALS patients, whereas proximal epidermal nerve fibers were at the lower end of the normal range of values reported for normal controls. In our previous study on small fiber neuropathy in ALS patients, a reduction of the distal INFD was found in subjects with spinal onset. Also abnormal laryngeal sensation was previously found in two studies by means of clinical assessment (8, 9). In our population, the group with laryngeal sensory deficit showed higher PAS scores, consistent with the increased difficulty to eject the material from the airway. The *P*-score values also indicated pathology, demonstrating the difficulties of ALS patients with laryngeal sensory deficit to handle the pooling in the hypopharynx.

The interaction of the motor and sensory deficits at the level of the aerodigestive crossroad is a serious condition that increases the risk of pneumonia, thus confirming the need for a detailed diagnostic instrumental workup to evaluate dysphagia. In particular, if the prevention of aspiration is no longer assured by the compensatory strategies, such as consistency modifications of foods and liquids and postures, the patient should undergo percutaneous endoscopic gastrostomy or radiologically inserted gastrostomy in accordance with the most recent European guidelines (23). Furthermore, from a clinical point of view, our data on laryngeal sensory deficits support FEES as the method of choice for swallowing evaluation in ALS subjects. Even if videofluoroscopy is more accurate in identifying subtle abnormalities in the pharyngeal phase of swallowing, FEES allows the assessment of the laryngeal sensitivity so critical in deciding the management of dysphagic patients. Furthermore, endoscopic examination is easy to perform, also at the ALS Center, avoiding patient discomfort, and it is repeatable at each subsequent control. In ALS, as in other neurodegenerative pathologies, the early detection of silent aspiration is critical to prevent such serious complications as pulmonary infections and malnutrition. In our sample, 4 out of the 62 patients who did not report swallowing problems showed abnormalities in the oral phases or sensitivity deficit of the larynx.

A limitation of our study is the small number of subjects available to undergo a laryngeal biopsy. Apart from the unwillingness of some patients to provide informed consent, this is due to the poor health conditions of the majority of ALS patients facing tracheotomy that usually discourage performing a subsequent laryngeal biopsy under microlaryngoscopy.

In conclusion, data from our study support sensory involvement in ALS patients. Sensory deficits at the level of the larynx were significantly more frequent in bulbar ALS. In three dysphagic ALS patients with a laryngeal sensory involvement, a biopsy of the laryngeal mucosa, showing in one subject no intraepithelial fiber and morphological changes in these fibers in the other two patients, allowed us to confirm the presence of a sensory deficit at the laryngeal level. All the patients with a deficit in laryngeal sensitivity also had oropharyngeal dysphagia. The combination of motor and sensory deficits at the level of the oropharynx leads to a serious dysphagia with an increased risk of aspiration. In all ALS patients, an early and accurate diagnostic workup to evaluate dysphagia is needed. FEES is the method of choice, allowing also the assessment of laryngeal sensitivity.

## Author Contributions

GR, AG, GC, MV, and MI: design of the study, interpretation of the data, preparation, and approval of the manuscript. EO and MG: analysis and interpretation of the data, preparation of the manuscript, review, and approval of the manuscript. IS, VF, MC, CC, and CB: collection and interpretation of the data, review and approval of the manuscript. AB and CG: immunohistochemical data analysis, review, and approval of the manuscript.

## Conflict of Interest Statement

The authors declare that the research was conducted in the absence of any commercial or financial relationships that could be construed as a potential conflict of interest.

## References

[1] Weis J Katona I Müller-Newen G Sommer C Necula G Hendrich C Small-fiber neuropathy in patients with ALS. Neurology (2011) 76(23):2024–9. 10.1212/WNL.0b013e31821e553a 21646630

[2] Pugdahl K Fuglsang-Frederiksen A de Carvalho M Johnsen B Fawcett PR Labarre-Vila A Generalised sensory system abnormalities in amyotrophic lateral sclerosis: a European multicentre study. J Neurol Neurosurg Psychiatry (2007) 78(7):746–9. 10.1136/jnnp.2006.098533 17575020 PMC2117695

[3] Hammad M Silva A Glass J Sladky JT Benatar M. Clinical, electrophysiologic, and pathologic evidence for sensory abnormalities in ALS. Neurology (2007) 69(24):2236–42. 10.1212/01.wnl.0000286948.99150.16 18071143

[4] Thompson RJ Doran JF Jackson P Dhillon AP Rode J PGP 9.5 a new marker for vertebrate neurons and neuroendocrine cells. Brain Res (1983) 278(1–2):224–8. 10.1016/0006-8993(83)90241-X 6640310

[5] Gulbenkian S Wharton J Polak JM The visualization of cardiovascular innervation in the guinea pig using an antiserum to protein gene product 9.5 (PGP 9.5). J Auton Nerv Syst (1987) 18(3):235–47. 10.1016/0165-1838(87)90122-6 3106456

[6] Lundberg LM Alm P Wharton J Polak JM. Protein gene product 9.5 (PGP 9.5). A new neuronal marker visualizing the whole uterine innervation and pregnancy-induced and developmental changes in the guinea pig. Histochemistry (1988) 90(1):9–17. 10.1007/BF00495700 2976412

[7] Truini A Biasiotta A Onesti E Di Stefano G Ceccanti M La Cesa S Small-fibre neuropathy related to bulbar and spinal-onset in patients with ALS. J Neurol (2015) 262(4):1014–8. 10.1007/s00415-015-7672-0 25683764

[8] Amin MR Harris D Cassel SG Grimes E Heiman-Patterson T. Sensory testing in the assessment of laryngeal sensation in patients with amyotrophic lateral sclerosis. Ann Otol Rhinol Laryngol (2006) 115(7):528–34. 10.1177/000348940611500707 16900807

[9] Ruoppolo G Schettino I Frasca V Giacomelli E Prosperini L Cambieri C Dysphagia in amyotrophic lateral sclerosis: prevalence and clinical findings. Acta Neurol Scand (2013) 128(6):397–401. 10.1111/ane.12136 23668293

[10] Hadjikoutis S Wiles CM Eccles R Cough in motor neuron disease: a review of mechanisms. QJM (1999) 92(9):487–94. 10.1093/qjmed/92.9.487 10627867

[11] Langmore SE Schatz K Olsen N Fiberoptic endoscopic examination of swallowing safety: a new procedure. Dysphagia (1988) 2(4):216–9. 10.1007/BF02414429 3251697

[12] Ruoppolo G Schettino I Biasiotta A Roma R Greco A Soldo P Afferent nerve ending density in the human laryngeal mucosa: potential implications on endoscopic evaluation of laryngeal sensitivity. Dysphagia (2015) 30(2):139–44. 10.1007/s00455-014-9589-7 25519304

[13] Rosenbek JC Robbins JA Roecker EB Coyle JL Wood JL A penetration-aspiration scale. Dysphagia (1996) 11(2):93–8. 10.1007/BF00417897 8721066

[14] Farneti D Fattori B Nacci A Mancini V Simonelli M Ruoppolo G The Pooling-score (*P*-score): inter- and intra-rater reliability in endoscopic assessment of the severity of dysphagia. Acta Otorhinolaryngol Ital (2014) 34(2):105–10. 24843220 PMC4025184

[15] Cedarbaum JM Stambler N Malta E Fuller C Hilt D Thurmond B The ALSFRS-R: a revised ALS functional rating scale that incorporates assessments of respiratory function. BDNF ALS study group (phase III). J Neurol Sci (1999) 169(1–2):13–21. 10.1016/S0022-510X(99)00210-5 10540002

[16] Gregson JM Leathley MJ Moore AP Smith TL Sharma AK Watkins CL. Reliability of measurements of muscle tone and muscle power in stroke patients. Age Ageing (2000) 29(3):223–8. 10.1093/ageing/29.3.223 10855904

[17] Lauria G Hsieh ST Johansson O Kennedy WR Leger JM Mellgren SI European Federation of Neurological Societies/Peripheral Nerve Society guideline on the use of skin biopsy in the diagnosis of small fiber neuropathy. Report of a joint task force of the European Federation of Neurological Societies and the Peripheral Nerve Society. Eur J Neurol (2010) 17(7):903–12. 10.1111/j.1468-1331.2010.03023.x 20642627

[18] Lauria G Morbin M Lombardi R Borgna M Mazzoleni G Sghirlanzoni A Axonal swellings predict the degeneration of epidermal nerve fibers in painful neuropathies. Neurology (2003) 61(5):631–6. 10.1212/01.WNL.0000070781.92512.A4 12963753

[19] Gibbons CH Griffin JW Polydefkis M Bonyhay I Brown A Hauer PE The utility of skin biopsy for prediction of progression in suspected small fiber neuropathy. Neurology (2006) 66(2):256–8. 10.1212/01.wnl.0000194314.86486.a2 16434668

[20] Herrmann DN McDermott MP Henderson D Chen L Akowuah K Schifitto G Epidermal nerve fiber density, axonal swellings and QST as predictors of HIV distal sensory neuropathy. Muscle Nerve (2004) 29(3):420–7. 10.1002/mus.10567 14981742

[21] Wendelschafer-Crabb G Kennedy WR Walk D. Morphological features of nerves in skin biopsies. J Neurol Sci (2006) 242(1–2):15–21. 10.1016/j.jns.2005.11.010 16448669

[22] Lauria G Holland N Hauer P Cornblath DR Griffin JW McArthur JC. Epidermal innervation: changes with aging, topographic location, and in sensory neuropathy. J Neurol Sci (1999) 164(2):172–8. 10.1016/S0022-510X(99)00063-5 10402030

[23] Andersen PM Abrahams S Borasio GD de Carvalho M Chio A Van Damme P EFNS guidelines on the clinical management of amyotrophic lateral sclerosis (MALS) – revised report of an EFNS task force. Eur J Neurol (2012) 19(3):360–75. 10.1111/j.1468-1331.2011.03501.x 21914052

